# The mutational burden in os odontoideum patients

**DOI:** 10.1186/s13023-026-04342-1

**Published:** 2026-04-14

**Authors:** Yinglun Tian, Guodong Gao, Dongwei Fan, Shilin Xue, Qiyue Gao, Cheng Zhang, Nanfang Xu, Shenglin Wang

**Affiliations:** 1 Department of Orthopaedics, Peking University Third Hospital, No 49. North Garden Road, HaiDian District, Haidian, Beijing, 100191 China; 2 Beijing Key Laboratory of Spinal Disease Research, Haidian, Beijing, 100191 China; 3 Engineering Research Center of Bone and Joint Precision Medicine, Ministry of Education, Haidian, Beijing, 100191 China

**Keywords:** Os odontoideum, Sequence analysis, Genetic variation, Genetic inheritance

## Abstract

**Background:**

Os odontoideum(OO) is a rare bone malformation at the craniovertebral junction, the presence of which can lead to potential instability of atlantoaxial joints. The cause, prevalence and treatment of OO are still controversial. There are two main theories regarding the pathogenesis of OO: congenital factors and traumatic factors. The congenital hypothesis suggests that the lesion results from segmental defects caused by incomplete fusion of the dens and the axis vertebral body during embryonic development. Our research team previously reported a case series involving three generations of a family affected by OO without a clear history of trauma, suggesting the existence of congenital pathogenic factors for this disease. Based on this, we conducted this study to further explore the pathogenesis of OO.

**Methods:**

We consecutively recruited 25 OO patients including 15 males(60%) and 10 females(40%) from 2021 to 2023. The clinical manifestation and concomitant deformities were analyzed and whole-exome sequencing(WES) was performed. The variants in OO patients were compared with those from 79 normal population controls using a case-control association analysis of individual rare variants. The analysis results were used to prioritize disease-associated candidate genes by combining Odds Ratio(OR) values and P values. OR values measure the strength of association between genetic variations and the disease, while P values determine the statistical significance of the results. Nominally high OR values and low P values can indicate a potential association between genetic variations and the disease. By integrating these two metrics, genes with notable correlations to OO were identified.

**Results:**

The analysis identified four genes nominally associated with OO based on their P values and OR values. Cell Division Cycle 27(CDC27) exhibited a P value of 0.002 and an OR of 5.08, indicating a notable association with the disease. Facioscapulohumeral Muscular Dystrophy Region Gene 1 Binding Protein(FRG1BP) showed a P value of 0.004 and an OR of 5.59, further supporting its potential relevance. Tripartite Motif Containing 8(TRIM8) had a P value of 0.02 and an OR of 4.58, suggesting a potential association. Centrosomal Protein 250(CEP250) demonstrated the most notable correlation among the four genes, with a P value of 0.005 and an OR of 7.78.

**Conclusion:**

Our study identifies rare variants potentially linked to OO and provides preliminary data that could inform hypotheses regarding a complex genetic basis, though larger cohorts and functional studies are needed for validation. Our findings of rare variants associated with OO, though preliminary, highlight a potential complex genetic basis for this condition. This underscores the need for further research which may ultimately aid in risk stratification and early detection in high-risk families.

## Introduction

Os odontoideum(OO) is a rare bone malformation at the craniovertebral junction, where the apex of the dens is separated from the main body of the odontoid process [1]. In 1886, Giacomini et al. first reported OO bone malformation [2]. The cause, prevalence and treatment of OO are still controversial [3, 4], and studies have shown that the prevalence of OO varies among different ethnic groups [5]. The origin of OO is primarily explained by two prevailing theories: congenital and traumatic [6, 7]. The congenital hypothesis suggests that the lesion results from segmental defects caused by incomplete fusion of the dens and the axis vertebral body during embryonic development. This failure is attributed to developmental issues at the synchondrosis between ossification centers [8, 9]. Additional mechanisms proposed under this theory include incomplete caudal migration of the axis centrum, segmentation failure, or nontraumatic osteonecrosis [10]. Supporting evidence for the congenital theory includes findings from patients with no history of trauma and the higher prevalence of OO among individuals with congenital malformations such as neurofibromatosis or skeletal dysplasia [11, 12]. Familial cases of OO exhibiting autosomal dominant inheritance patterns, as well as reports of OO in identical twins, provide additional support for this hypothesis [13–17]. Radiological studies also reinforce the congenital etiology, with the identification of a joint between the odontoid and the anterior arch of the atlas, termed the “jigsaw sign,” as a reliable indicator of congenital OO [18, 19].

The embryonic origin of the odontoid process is from three sclerotomes (the fourth occipital and the first and second cervical sclerotome) [20, 21]. The cephalad tip of the odontoid process derives from the fourth occipital sclerotome [21] and is termed ossiculum terminale or apical odontoid epiphysis, which normally appears by three to six years of age and fuses with the main body of the odontoid by twelve years of age [22]. The main body of the odontoid process is from the first cervical sclerotome, while the centrum derives from the second cervical sclerotome [8]. The condition was first identified by Giacomini and has since been categorized into two types: orthotopic and dystopic [18]. The orthotopic type refers to an ossicle that moves in coordination with the anterior arch of C1, whereas the dystopic type describes an ossicle that is structurally fused to the basion [23].

**Fig. 1:**
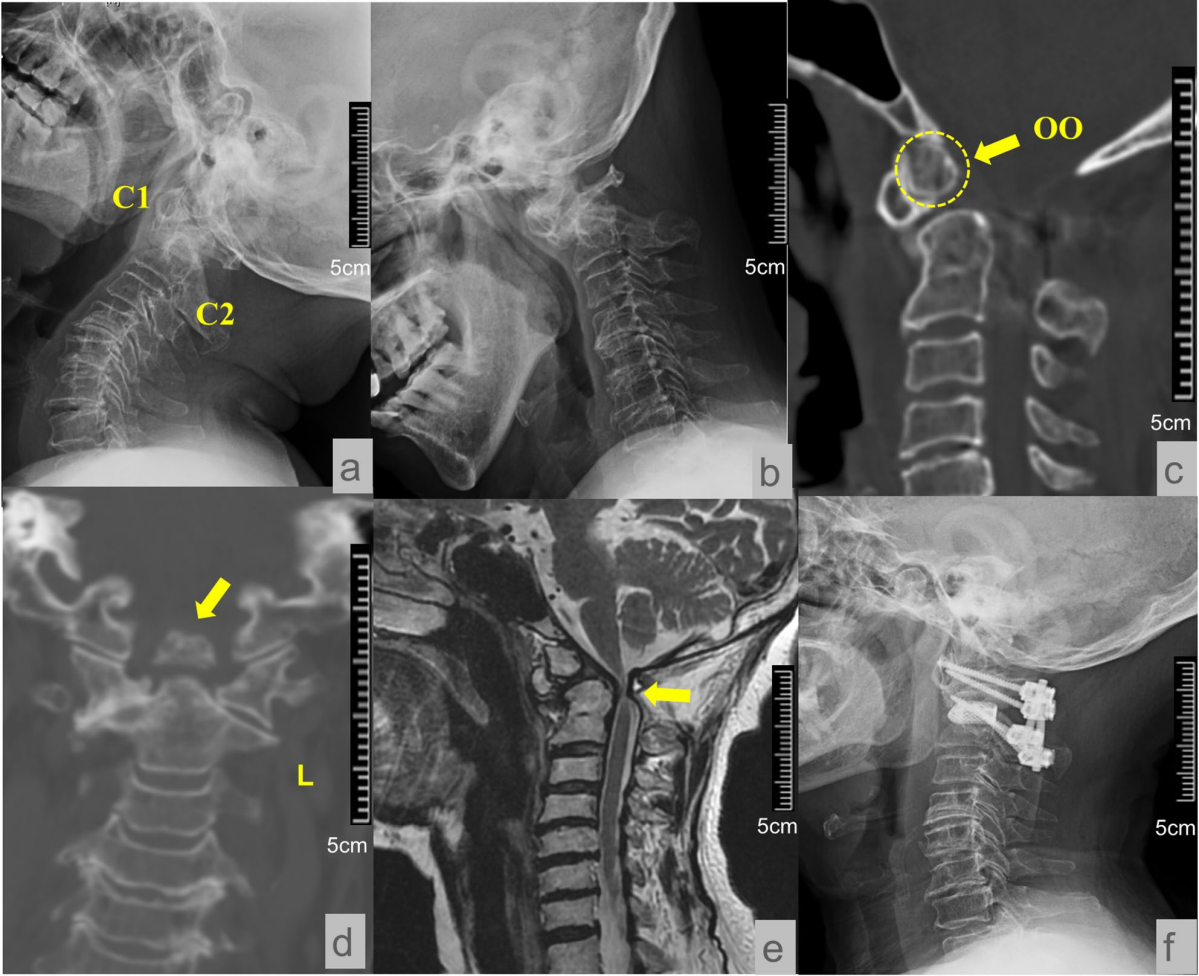
A 76-y/o female OO patient. **a**&**b.** preoperative dynamic X-ray showed OO deformity and atlantoaxial dislocation; **c.** preoperative CT with sagittal images showed OO deformity(within the dashed circle): the apex of the odontoid process is separated from the base of the C2 vertebral body; **d.** preoperative CT with coronal images showed OO deformity(yellow arrow); **e.** preoperative MRI showed the severe compression of the spinal cord: the spinal cord is thinned(yellow arrow); **f.** postoperative X-ray showed that the atlantoaxial joint was reduced and fixed using screws, and a cage was placed between the atlantal and axial lateral masses for stabilization

The presence of OO can lead to potential instability of atlantoaxial joints. Once atlantoaxial dislocation (AAD) occurs, the atlantoaxial joints lose their normal involvements, which may lead to spinal cord compression [19, 24, 25](Fig. 1). Severe compression can sometimes affect vertebral arterial blood flow, leading to intracranial diseases such as cerebellar infarction or brain stem injury [26–28]. The clinical manifestations of OO patients are varying in severity from no symptom to dizziness, headache and quadriplegia, which even affect the respiratory center leading to respiratory failure and death [29, 30].

However, the presence of OO bone malformation in craniovertebral junction will undoubtedly increase the difficulty of operational treatment. Goel’s study [25] found that OO patients had a significantly higher ratio of high-riding vertebral artery, so it was important to avoid vertebral artery injury during the operation. How to accomplish the early diagnosis and do non-surgical intervention of OO bone malformation is an urgent problem in the clinical practice, and the in-depth exploration of its pathogenesis is the basis for the early diagnosis and non-surgical intervention. To investigate the potential genetic susceptibility factors underlying the congenital pathogenesis of OO, we performed whole exome sequencing(WES) on a cohort of 25 patients without clear trauma history. We aimed to identify rare variants associated with OO and preliminarily explore genotype-phenotype correlations.

## Materials and methods

Institutional Review Board approval was obtained before the initiation of this study(M2022344). The patients who were diagnosed as OO in Peking University Third Hospital were prospectively included from 2021 to 2023. This group of patients included 25 OO patients, 15 males(60%) and 10 females(40%) with an age distribution of 13–76 years(mean 53.1 years). Cervical stability was assessed using the Atlanto-Dental Interval (ADI) on lateral radiographs. Neurological status was evaluated using the Japanese Orthopaedic Association (JOA) score. The presence of spinal cord high signal intensity was determined by preoperative T2-weighted MRI at the craniovertebral junction.

An approved consent form was signed by each patient before any testing was performed. Their clinical manifestation and concomitant deformities were gathered. The diagnosis of OO was based on the plain radiographic and computed tomography(CT) scan evidence of an ossicle with smooth circumferential cortical margins separated from the body of the axis. Patients were excluded in the presence of any of the following scenarios:

1. Patients with a history of an odontoid fracture or with radiographic evidence of an existing odontoid fracture.
2. Cases of isolated ossiculum terminale, which represents only the very cephalad portion of the odontoid process, resulting from the incomplete fusion of the apex at the secondary ossification center [31]. Unlike OO, ossiculum terminale is characterized by a smaller ossicle and is rarely linked to C1-C2 instability. Moreover, it typically does not necessitate surgical intervention [29, 31].

Whole exome sequencing (WES) was performed on peripheral blood DNA collected from all patients, with 1–3 µg DNA extracted from each sample. The DNA was fragmented to an average size of 180 bp using ultrasound sonication and paired-end sequencing libraries were constructed. Targeted libraries with an insert size of 150–200 bp were purified using Solid Phase Reverse Immobilization (SPRI) magnetic beads and sequenced on the Illumina HiSeq X platform, generating paired-end reads of 150 bp. The mean sequencing coverage depth was > 100×, with > 95% of the target regions covered at ≥ 20×. Base quality distribution quality control was performed to ensure high-quality sequencing data.

**Fig. 2:**
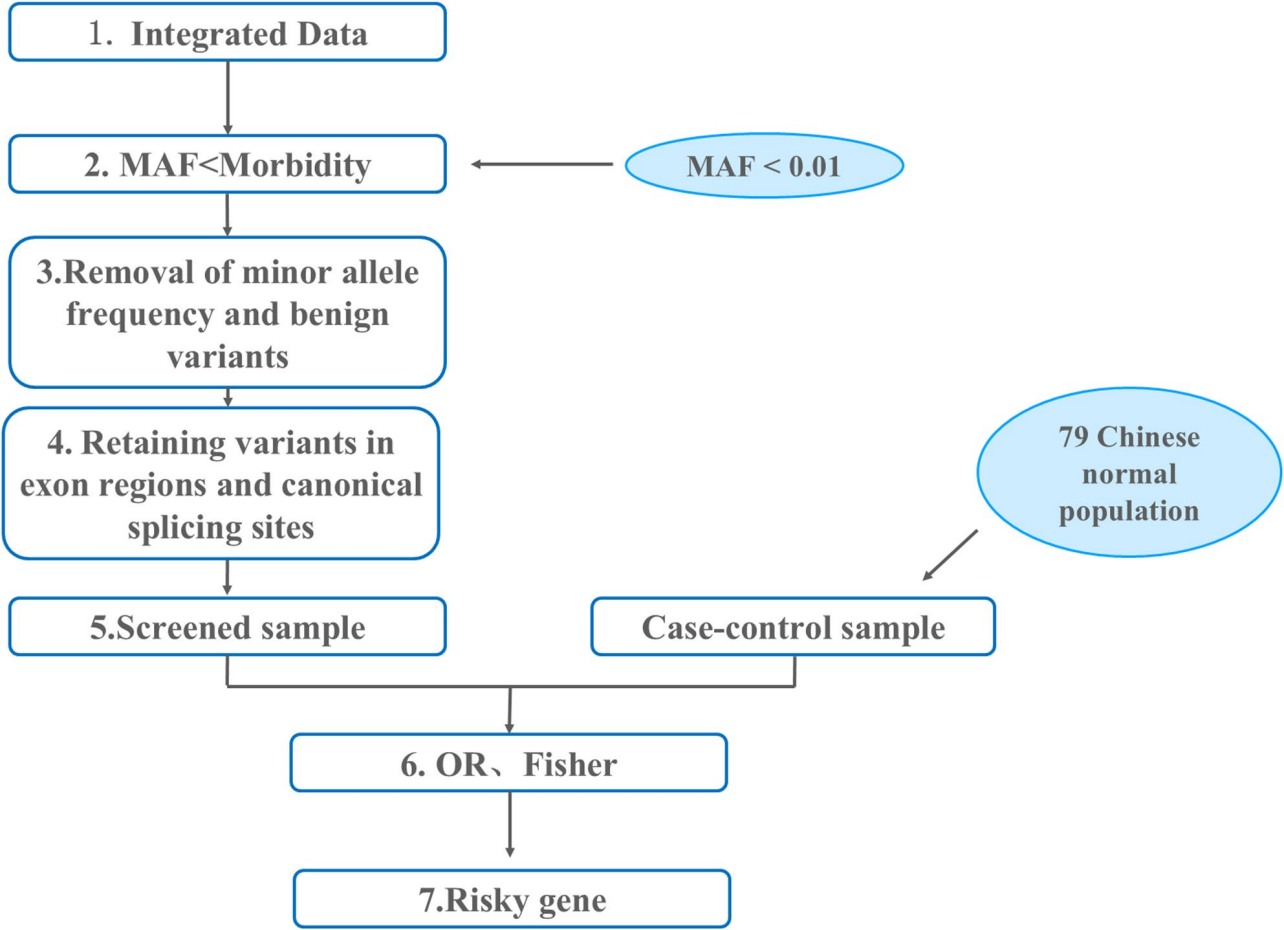
Data processing algorithm

First, variable filtering was conducted according to the following principles(Fig. 2):

(i) Exclusion of variants with a minor allele frequency (MAF) greater than 1% (MAF > 0.01) in the East Asian (EAS) population of the gnomAD database;
(ii) Removal of benign variants annotated in ClinVar;
(iii) Exclusion of single nucleotide polymorphisms (SNPs) and untranslated regions (UTR) within introns;
(iv) Retaining rare exonic and canonical splice-site (± 1,2) variants, excluding synonymous changes. Additionally, non-coding variants (e.g., in UTRs) predicted by bioinformatics tools (ESE Finder 3.0) to have potential regulatory functions were also retained for evaluation.

Following the four-step filtering process, low-quality sequences (< 80 bp) were excluded using Cutadapt, and high-quality reads were aligned to the human reference genome (hg19) using BWA. Duplicate sequences were removed with Picard, and variation detection was performed using GATK Haplotype Caller. Variants were further filtered using GATK Variant Filtration based on the following criteria: (a) Mapping Quality (MQ) < 30; (b) Total MQ Zero Reads < 4; (c) Approximate Read Depth < 5; (d) QUAL < 50.0; and (e) phred-scaled p-value > 10.0 (Fisher’s Exact Test for strand bias). Annotated variants were analyzed using ANNOVAR, incorporating multiple databases such as 1000 Genome, ESP6500, dbSNP, ExAC, HGMD, and ClinVar. Pathogenic predictions for filtered variants were performed using SIFT, PolyPhen-2, MutationTaster, and GERP++, while conservation analysis of mutant transcripts was conducted across species using CLUSTAL W and UGENE. For non-coding region variants, functional predictions were made using ESE Finder 3.0.

After filtering, the data were compared with WES test results from 79 Chinese healthy individuals to perform a case-control association analysis of individual rare variants. Specifically, we evaluated the frequencies of specific qualifying rare variants (MAF < 0.01 in gnomAD EAS, primarily exonic/canonical splice-site or functionally predicted UTRs) across the cohorts. For each specific variant, we compared the proportion of carrier individuals between the OO cases and controls. Odds ratios (OR) and P-values were calculated using Fisher’s exact test, and 95% confidence intervals (CI) were generated. A total of 420 qualifying rare variants were evaluated in this screening phase. Potential candidate genes associated with these variants were further investigated based on annotations from the NCBI gene database (https://www.ncbi.nlm.nih.gov/gene) and the Human Protein Atlas (HPA, https://www.proteinatlas.org).

## Results

### Patient characteristics and clinical associations

This group of patients all had os odontoideum (OO) bone malformations. Among the 25 patients, two exhibited additional anomalies. One patient presented with C2-4 fusion, absence of the posterior atlas arch, nonunion of the anterior atlas arch, and C5 hemivertebra, while another patient had C1 occipitalization. However, neither of these patients demonstrated a distinct type of OO bone malformation. Their clinical manifestations were not more severe compared to other patients. Both patients retained full mobility in their limbs, and the atlantoaxial dislocations caused by OO were reducible. The remaining 23 patients exhibited only OO deformities (Table 1).

**Table 1:** The clinical characteristics of the OO cohort

Parameter	OO corhort(*n* = 25)
**Age**,** mean(range)**	53.1(13–76)
**Sex**,** n(%)**	
Men	15(60%)
Women	10(40%)
**Combined deformities**,** n(%)**	
OO	25(100%)
Klippel-Feil Syndrome	1(4%)
Absence of posterior atlas arch	1(4%)
C1 occipitalization	1(4%)
**Treatment Received**	
Posterior atlantoaxial reduction and fixation, n (%)	25(100%)
**Genotype-Phenotype Correlation (by Number of Candidate Mutations Carried)**	
**0 Mutations (n = 6)**	
Preoperative ADI (mm), mean (range)	9.80(7.1–13.3)
Preoperative JOA score, mean (range)	15.83(15–17)
Spinal cord high signal present, n (%)	5(83.3%)
**1 Mutation (n = 8)**	
Preoperative ADI (mm), mean (range)	5.47(3.0–10.0)
Preoperative JOA score, mean (range)	12.75(9–16)
Spinal cord high signal present, n (%)	8(100%)
**2 Mutations (n = 11)**	
Preoperative ADI (mm), mean (range)	8.39(3.0-14.1)
Preoperative JOA score, mean (range)	14.45(13–17)
Spinal cord high signal present, n (%)	11(100%)

**OO-os odontoideum**

**Table 2:** The case-control association analysis for selected rare variants in OO cases and controls

	OO cases(*n* = 25)	Control subjects(*n* = 79)	*p*-value	OR	95%CI	Bonferroni P*
CDC27	10	9	0.002	5.08	(1.78–14.50)	0.84
FRG1BP	8	6	0.004	5.59	(1.72–18.21)	1.68
TRIM8	6	5	0.02	4.58	(1.26–16.63)	8.4
CEP250	6	3	0.005	7.78	(1.81–33.51)	2.1

*Note: Bonferroni-corrected P-values are based on the 420 variants tested in this screening phase

**Table 3:** Detailed annotation for the four identified rare variants

	Variant nomenclature	MAF	CADD Score	pLI Score
CDC27	c.1795G > A(*p*.A599T)	0	8.456	0.99
FRG1BP	c.1-8028T > C	0	1.362	0.00
TRIM8	c.814 C > T(p.L272F)	0	5.505	0.99
CEP250	c.665G > A(p.R222H)	0	11.88	0.00

### Statistical tests and genetic case-control association analysis

For statistical analysis, the study employed a case-control association analysis to evaluate the association between identified variants and OO. Odds ratios (OR) and P-values were calculated based on Fisher’s exact test. Given the relatively small sample size and the exploratory nature of the study, no multiple comparison corrections (e.g., Bonferroni or FDR) were applied. Instead, the focus was placed on identifying variants with strong statistical significance and biological plausibility. This approach is widely used in genetic studies of rare diseases, where sample sizes are often limited, and the goal is to prioritize candidate genes for further investigation. To provide transparency, we report the nominal P-values alongside Bonferroni-corrected P-values (based on testing 420 variants) in the footnote of Table 2, acknowledging that none survive strict correction.

Through this analysis, combined with the evaluation of P-values and OR from the final dataset, four genes were identified as potentially associated with OO. These include Cell Division Cycle 27 (CDC27), which displayed a P-value of 0.002 and an OR of 5.08, suggesting a notable correlation; Facioscapulohumeral Muscular Dystrophy Region Gene 1 Binding Protein (FRG1BP), with a P-value of 0.004 and an OR of 5.59, further reinforcing its potential role; Tripartite Motif Containing 8 (TRIM8), showing a P-value of 0.02 and an OR of 4.58, indicating a potential association; and Centrosomal Protein 250 (CEP250), with a P-value of 0.005 and an OR of 7.78, representing the most notable correlation among the four (Table 2).

### Gene variants and biological mechanisms

Through the case-control association analysis of individual rare variants, specific genetic variations were identified in several patients, highlighting potential associations with the disease. Specifically, the CDC27 variant (NM_001114091.4: exon14: c.1795G > A: p.A599T) was detected in 10 patients, while this specific variant was found in only 9 out of 79 control subjects, yielding a nominal P-value of 0.002 and an OR of 5.08 (95% CI: 1.78–14.50).

Similarly, the FRG1BP variant (NM_003579.2: exon4: c.1-8028T > C), which is located in the 5’-UTR but was retained due to its prediction by ESE Finder 3.0 as potentially affecting splicing, was observed in 8 patients, compared to 6 control subjects, with a P-value of 0.004 and an OR of 5.59 (95% CI: 1.72–18.21).

Furthermore, the specific missense mutation in CEP250 (NM_007186.6: exon9: c.665G > A: p.R222H) and the variant in TRIM8 (NM_030912.2: exon3: c.814 C > T: p.L272F) were found in 6 patients each, while these variants appeared in 3 and 5 control subjects, respectively. The P-values for these individual variants were 0.005 (CEP250) and 0.02 (TRIM8), with ORs of 7.78 (95% CI: 1.81–33.51) and 4.58 (95% CI: 1.26–16.63), respectively. (Tables 2 and 3).

To enhance the biological interpretation of these findings, we explored the functional roles of the identified genes. CDC27 is involved in cell cycle regulation and may affect bone development through dysregulation of mitotic processes [32, 33]. FRG1BP has been implicated in muscular dystrophy and could play a role in musculoskeletal development [34, 35]. TRIM8 is associated with inflammatory pathways, which might contribute to the pathogenesis of OO [36, 37]. CEP250 is a centrosomal protein critical for microtubule organization, which could influence skeletal morphogenesis [38]. These potential mechanisms are further discussed in the Discussion section.

### Descriptive genotype-phenotype correlation

To explore the relationship between genetic findings and clinical severity, patients were categorized into three groups based on the number of candidate mutations identified (0, 1, or 2). Among the 25 OO patients, 6 (24%) carried no mutations in the four candidate genes, 8 (32%) carried one mutation, and 11 (44%) carried two mutations. The mean preoperative ADI for the 0, 1, and 2-mutation groups was 9.8 mm, 5.47 mm, and 8.39 mm, respectively. Regarding neurological function, the mean preoperative JOA scores were 15.83, 12.75, and 14.45, respectively. Additionally, preoperative MRI revealed high signal changes in the spinal cord at the craniovertebral junction in 24 of the 25 patients (96%), with only one patient showing no such signal alteration(Table 1).

Given the near-universal presence of spinal cord high signals in this cohort, and the non-linear distribution of ADI and JOA scores across the mutation groups, these descriptive observations do not support a simple linear dosage effect, suggesting that the relationship between mutation burden and clinical severity may be complex and non-linear. This descriptive analysis highlights the complex and highly heterogeneous phenotypic expression of the disease, which may not be fully explained by a simple cumulative genetic dosage model.

## Discussion

OO is a rare bone deformity of the craniovertebral junction. The presence of this deformity can lead to atlantoaxial dislocation, which compresses the medulla and spinal cord, resulting in a significant risk of disability and mortality. Currently, effective treatment is only achievable through surgical intervention, but the timing of surgery typically occurs after patients present with symptoms and are diagnosed. In some cases, neurological damage has already become irreversible by the time of diagnosis. Therefore, early diagnosis and timely treatment for OO patients are particularly crucial. The pathogenesis of OO remains unclear, and the associated genetic variations are also uncertain. Based on the genetic heterogeneity observed in OO patients in clinical practice, we aim to conduct this study to identify genetic variation evidence in OO patients.

### The pathogenesis of os odontoideum

As for the two theories about the incidence of OO: congenital factors and traumatic factors [6]. The traumatic theory suggested that OO was caused by the pathological process of avascular necrosis resulted from odontoid fracture. The traumatic theory was also widely supported by reports of OO cases with a history of trauma [39], but some groups of familial OO or twin OO cases with no history of trauma appeared to provide evidence for the theory of innate causes of OO. Morgan [14] has reported on a 16-year-old male patient with asymptomatic OO malformation, whose family screening found that both his father and grandfather had the same OO bone malformation. Stevens [40] has reported a group of OO cases involving three generations of the family. They believed that the onset characteristics of OO in the family conformed to the genetic rule of autosomal dominant diseases. Kirlew [39] reported a case of identical twin sisters with the same OO deformity. Al Kaissi [41] has also reported on a group of OO siblings. And our team also have reported a group of OO cases involving three generations of the family with no clear history of trauma in 2011 [42].

Although the specific pathogenic genes for OO remain unidentified, genetic studies have revealed significant gene expression differences associated with the condition. For instance, CMK4, ATF1, PLCG1, Table 1, E2F3, and ATF4 showed altered expression in twin OO cases compared to non-twin OO cases, while MMP8, KIT, HIF1A, CREB3, PWHAZ, and TGFBR1 were differentially expressed in OO patients compared to non-OO individuals [16, 43]. These findings suggest potential involvement of pathways related to bone development, cell cycle regulation, and inflammatory responses. Familial and twin studies further support the hypothesis of autosomal dominant inheritance in OO, providing a genetic basis for its congenital origin. However, given the clinical heterogeneity we observed—where some patients presented with additional anomalies like C1 occipitalization while others had isolated OO—it is likely that multiple genetic factors or mechanisms contribute to the phenotype.

### The related mechanisms of target genes

 To assess the plausibility of our candidate genes, we evaluated variant-level metrics including population frequency (gnomAD), deleteriousness (CADD), and gene constraint (pLI)(Table 3). Previous studies have reported that FRG1 was a candidate gene of Facioscapulohumeral Muscular Dystrophy(FSHD) [44]. In our study, the FRG1BP variant (c.1-8028T > C) is located in the 5’-UTR. While our primary filtering aimed for exonic variants, this specific site was retained because ESE Finder 3.0 predicted it could influence splicing enhancers, potentially affecting the blood supply of the odontoid process. Using the development model of African toad, Ryan et al. [45] demonstrated that FRG1 mutation would lead to abnormal development of muscle tissue, and FRG1 expression was found to be crucial for vascular development. The down regulation of FRG1 would lead to decreased angiogenesis and decreased expression of the angiogenesis regulator DAB2. As the family gene of FRG1, could the FRG1BP variation is related to the blood supply of odontoid process? Aberrant expression of FRG1 has been linked to improper skeletal and muscular development, suggesting its potential role in bone formation and ossification processes [34, 35]. While its direct involvement in odontoid development remains unclear, FRG1BP may influence the formation and fusion of ossification centers through its regulatory effects on musculoskeletal interactions.

CDC27, a core component of the anaphase-promoting complex/cyclosome (APC/C), plays a critical role in regulating the cell cycle, particularly during mitosis. The CDC27 variant (p.A599T) identified in our cohort is entirely absent in the gnomAD EAS population (MAF = 0). Although its CADD score of 8.46 and low pLI score suggest it is not a highly penetrant loss-of-function mutation, its ultra-rare nature points to its potential functional importance as a subtle disease modifier. Its involvement in skeletal development is suggested through its regulation of chondrocyte proliferation and differentiation, processes essential for endochondral ossification. Studies have shown that APC/C-mediated regulation is vital for proper skeletal growth and development, as disruptions in cell cycle control can lead to developmental defects in bone tissue [32, 46]. Other studies have found that the abnormal expression of CDC27 was related to cell proliferation, migration, secretion and other functions, and was closely related to the occurrence and development of pituitary stem interruption syndrome, tumor and other diseases [47]. These findings imply that mutations in CDC27 could impair the proliferation and maturation of chondrocytes, potentially contributing to abnormalities such as os odontoideum.

TRIM8, an E3 ubiquitin ligase, has been shown to modulate inflammatory pathways, including the NF-κB signaling pathway, which plays a significant role in bone remodeling and repair. Similarly, the identified TRIM8 variant (p.L272F) is notably absent in the gnomAD EAS population (MAF = 0) and exhibits a modest CADD score of 5.51, suggesting it may modestly impact the protein’s conserved domain without causing lethal developmental arrest. Dysregulation of TRIM8 has been associated with altered cellular responses to inflammatory stimuli, which may impact the homeostasis of bone tissue [36, 37]. Recent studies on TRIM8 strongly advocate for the critical role of TRIM8 as a regulator of cell proliferation [48] and as an important player in cancer [49], immunity [50], and inflammation [51]. Studies have shown that TRIM8 regulated cell cycle progression and mitosis by affecting cell cycle checkpoints and critical mitotic regulators and indirectly interacts with various motor microtubule-associated proteins (MAPs) such as kinesins [52]. Given the involvement of inflammation in the pathogenesis of skeletal abnormalities, TRIM8 mutations could contribute to os odontoideum by disrupting the balance between bone resorption and formation during development.

CEP250, a centrosomal protein, is critical for microtubule organization and the maintenance of cytoskeletal stability [53]. The CEP250 variant (p.R222H) not only showed the strongest nominal association in our study (OR = 7.78), but also possessed the highest predicted deleterious score among the four variants (CADD = 11.88), further supporting its biological plausibility despite its ultra-rare prevalence (MAF = 0). CEP250 is a key regulator of centrosome cohesion, which connects the two parent centrioles after their duplication [54]. This connection is established through a proteinaceous linker containing rootlet in (CROCC) and LRRC45 filaments [55]. Recently, a truncating mutation of Cep250 in cattle has been associated with the Caprine-like Generalized Hypoplasia Syndrome, due to the loss of centrosome cohesion and centrosome splitting [56]. This syndrome associates with features similar to those of autosomal recessive primary microcephaly (MCPH) and Seckel-like syndrome. In humans, CEP250 mutations are associated with Usher syndrome [57]. Mutations in CEP250 have been linked to developmental abnormalities, including defects in skeletal morphogenesis [38]. As centrosomes are essential for proper cell division and spatial organization during bone growth, disruptions in CEP250 function may impair the fusion of ossification centers, leading to conditions such as os odontoideum. Furthermore, its role in regulating cellular architecture highlights its importance in maintaining the structural integrity of developing skeletal tissues.

It is noteworthy that while the variants presented in our study are ultra-rare (e.g., absent in the gnomAD EAS database), their predicted deleterious scores (CADD scores ranging from 1.36 to 11.88) are relatively modest compared to typical highly penetrant Mendelian disease-causing mutations. However, this perfectly aligns with the clinical phenotype of our cohort. Os odontoideum patients in our study were primarily adults (mean age 53.1 years) without severe, early-onset syndromic or lethal manifestations. Therefore, we hypothesize that these variants act as moderate-effect genetic susceptibility factors rather than fully penetrant causative mutations. (Furthermore, the low CADD score for FRG1BP is expected, as it is located in the 5’-UTR where CADD algorithms are notably less sensitive, yet alternative tools like ESE Finder predicted potential splicing alterations).

Because these variants likely act as moderate-effect susceptibility factors, the etiology of OO is unlikely to be fully explained by a simple monogenetic model. Indeed, our descriptive genotype-phenotype analysis revealed that patients carrying more candidate mutations did not necessarily exhibit more severe radiographic instability or neurological symptoms. This lack of a strict linear ‘dosage effect’ strongly suggests that OO is a highly heterogeneous condition. The clinical phenotype is likely influenced not only by the mere number of mutations but also by the specific functional impact of each variant, compensatory anatomical mechanisms, and environmental factors. While we observed some patients carrying multiple variants across these candidate genes, our current dataset is underpowered to formally confirm an oligogenic inheritance pattern. However, it is plausible to hypothesize that the combinatorial effects of these rare variants in interacting biological pathways modify the phenotype of OO through complex synergistic or counteracting effects. Therefore, the results of this study should be strictly interpreted as hypothesis-generating. This complex interplay between genetic susceptibility and anatomic biomechanics warrants further investigation in larger, multicenter cohorts and functional models.”

### Limitations

The limitations of this study primarily include three aspects. First, as OO is a rare disease, the sample size is relatively small, which limited our statistical power and prevented the meaningful application of multiple-comparison corrections. The identified associations are nominal and require replication. Second, since most patients are diagnosed after the age of 50, many of their parents have already passed away, which has prevented us from obtaining genetic testing results from OO-related families to perform segregation analysis. Third, while bioinformatics tools suggest potential pathogenicity, functional validation of these specific variants in bone development models is currently lacking. This will be the focus of our future research.

## Conclusions

In conclusion, our study represents an important step in investigating the genetic basis of OO, providing a detailed overview of the clinical characteristics and WES findings in this patient cohort. Through case-control association analysis, we identified four rare variants in CDC27, FRG1BP, TRIM8, and CEP250 that are nominally associated with OO, offering preliminary insights into the genetic factors contributing to this condition. While our findings do not confirm a specific inheritance pattern, they provide prioritized candidates for future research. Future studies with larger cohorts and functional experiments are needed to confirm these associations and clarify their biological significance.

Our study adds to the limited genetic data on OO by employing a case-control WES approach, further comparisons with existing literature are required to substantiate this claim. Leveraging the advantage of our center’s extensive experience with atlantoaxial disease cases, we plan to expand the sample size in future research. Our ultimate goal is to provide stronger evidence to elucidate the pathogenesis of OO, enabling earlier diagnosis and more effective treatment strategies. By identifying potential genetic biomarkers, we move closer toward a personalized approach to managing this complex craniovertebral deformity.

## Data Availability

The data that support the findings of this study are not openly available due to reasons of sensitivity and are available from the corresponding author upon reasonable request.

## Abbreviations

OO: Os odontoideum
AAD: atlantoaxial dislocation
OR: odds ratios
WES: whole exome sequencing
CDC27: Cell Division Cycle 27
FRG1BP: Facioscapulohumeral Muscular Dystrophy Region Gene 1 Binding Protein
TRIM8: Tripartite Motif Containing 8
CEP250: Centrosomal Protein 250
